# Mutational Profile and Pathological Features of a Case of Interleukin-10 and RGS1-Positive Spindle Cell Variant Diffuse Large B-Cell Lymphoma

**DOI:** 10.3390/hematolrep15010020

**Published:** 2023-03-12

**Authors:** Joaquim Carreras, Yara Yukie Kikuti, Masashi Miyaoka, Shinichiro Hiraiwa, Sakura Tomita, Haruka Ikoma, Yusuke Kondo, Atsushi Ito, Shunsuke Nagase, Hisanobu Miura, Giovanna Roncador, Lluis Colomo, Rifat Hamoudi, Elias Campo, Naoya Nakamura

**Affiliations:** 1 Department of Pathology, School of Medicine, Tokai University, 143 Shimokasuya, Isehara 259-1193, Kanagawa, Japan; 2 Monoclonal Antibodies Unit, Spanish National Cancer Research Center (Centro Nacional de Investigaciones Oncologicas, CNIO), Melchor Fernandez Almagro 3, 28029 Madrid, Spain; 3 Department of Pathology, Hospital del Mar, Passeig Maritim 25-29, 08003 Barcelona, Spain; 4 Department of Clinical Sciences, College of Medicine, University of Sharjah, Sharjah P.O. Box 27272, United Arab Emirates; 5 Division of Surgery and Interventional Science, University College London, Gower Street, London WC1E 6BT, UK; 6 Department of Pathology, Hospital Clinic Barcelona, August Pi i Sunyer Biomedical Research Institute (IDIBAPS), Esther Koplowitz Center (CEK), Centro de Investigacion Biomedica en Red de Cancer (CIBERONC), 08036 Barcelona, Spain

**Keywords:** diffuse large B-cell lymphoma, spindle cell, regulator of G protein signaling 1, interleukin 10, next-generation sequencing, mutational profiling, LymphGen 1.0

## Abstract

Diffuse large B-cell lymphoma with spindle cell morphology is a rare variant. We present the case of a 74-year-old male who initially presented with a right supraclavicular (lymph) node enlargement. Histological analysis showed a proliferation of spindle-shaped cells with narrow cytoplasms. An immunohistochemical panel was used to exclude other tumors, such as melanoma, carcinoma, and sarcoma. The lymphoma was characterized by a cell-of-origin subtype of germinal center B-cell-like (GCB) based on Hans’ classifier (CD10-negative, BCL6-positive, and MUM1-negative); EBER negativity, and the absence of *BCL2*, *BCL6*, and *MYC* rearrangements. Mutational profiling using a custom panel of 168 genes associated with aggressive B-cell lymphomas confirmed mutations in *ACTB*, *ARID1B*, *DUSP2*, *DTX1*, *HLA-B*, *PTEN*, and *TNFRSF14*. Based on the LymphGen 1.0 classification tool, this case had an ST2 subtype prediction. The immune microenvironment was characterized by moderate infiltration of M2-like tumor-associated macrophages (TMAs) with positivity of CD163, CSF1R, CD85A (LILRB3), and PD-L1; moderate PD-1 positive T cells, and low FOXP3 regulatory T lymphocytes (Tregs). Immunohistochemical expression of PTX3 and TNFRSF14 was absent. Interestingly, the lymphoma cells were positive for HLA-DP-DR, IL-10, and RGS1, which are markers associated with poor prognosis in DLBCL. The patient was treated with R-CHOP therapy, and achieved a metabolically complete response.

## 1. Introduction

Diffuse large B-cell lymphoma (DLBCL) is one of the most frequent non-Hodgkin lymphomas (NHL). According to the 2016 revision of the World Health Organization classification of lymphoid neoplasms, DLBCL accounts for around 25% of adult NHL cases [1]. Like most other NHLs, DLBCL is characterized by male predominance, the incidence increases with age, and it has a median age at presentation of 64 years [1,2,3,4,5,6]. Usually, the patients present with an enlarging symptomatic mass, nodal enlargement in the neck or abdomen, and advanced stage in 60% of cases. In up to 40% of cases, the disease arises from extranodal extramedullary tissues [1,2,3,4,5,6,7].

The diagnostic category of DLBCL is heterogeneous. The cases show heterogeneous characteristics regarding the morphological features, genetic alterations, and biological background [1,3]. DLBCL arises from mature B-lymphocytes, allegedly from the germinal centers of lymphoid follicles or from a post-germinal differentiation stage [5]. The molecular pathogenesis is complex and involves a multistep process involving aberrant expression; dysregulation; translocation; and/or mutations of *BCL2*, *BCL6*, and *MYC* [8]; *TP53* mutations; aberrant somatic hypermutations; immune evasion; abnormal lymphocyte trafficking; and cell-of-origin molecular subtypes [9].

The typical morphology shows effacement of the normal architecture lymph nodes or the extranodal tissues by tumor cells that are large and resemble normal centroblasts and immunoblasts [1]. Centroblasts are large, non-cleaved cells with a round or oval nucleus and multiple peripheral nucleoli. Immunoblasts are larger cells with very prominent nucleoli and cytoplasms, often with plasmacytoid characteristics [9].

There are other histological morphologies in addition to the centroblastic and immunoblastic, such as the anaplastic variant. This morphology is characterized by large bizarre pleomorphic cells in a cohesive or sheet-like growth. Another infrequently recognized variant is the multilobated [10]. Despite the fact that the prognostic value of the morphological variants is controversial due to reproducibility, we have recently described that a multilobated morphology of DLBCL is associated with a favorable prognosis of the patients [11]. Another unusual variant is the spindle cell morphology [12,13,14]. Since this variant is extremely infrequent, can be easily confused with other spindle cell tumors such as sarcomas or carcinomas. Therefore, the recognition of this morphological variant is worth reporting.

## 2. Materials and Methods

### 2.1. Histological Procedures

Histological analysis consisted of hematoxylin and eosin (H&E), Masson trichrome stain, immunohistochemical staining, and DNA in situ hybridization (FISH) with brake apart probes for *BCL2*, *BCL6*, and *MYC* (Vysis, Tokyo, Japan) [15,16,17]. Immunohistochemistry was performed following the manufacturer’s instructions (Leica Bond-Max autostainer, Leica Biosystems K.K., Tokyo, Japan) [18] and included a battery of primary antibodies as actin-SM, ALK-1, BCL2, BCL6, BTK D3H5, CD10, CD163, CD20 (L26), CD21, CD3, CD31, CD34, CD5, CD68, CD85A, CDK4, CK AE1/3, C-MYC, CSF1R, desmin, FOXP3, HLA-DP-DR, IL-10, MDM2, MIB-1, MUM1, p16, PD-1, PD-L1, PTEN, PTX3, RGS1, S100, and TNFRSF14 (HVEM). The primary antibodies were purchased or obtained from Novocasra (Leica), ThermoFisher, Perseus Proteomics, Abcam, and the Spanish National Cancer Research Center (CNIO). Immunofluorescence was performed as described previously [18,19]. The slides were observed using an optical BX51 Olympus microscope, scanned using a Zeiss LSM700 confocal microscope, and visualized with Imaris Bitplane 3D rendering software (Oxford Instruments, Abingdon, Oxfordshire, UK) [18,20].

### 2.2. Targeted Next-Generation Sequencing

DNA was extracted from formalin-fixed paraffin-embedded (FFPE) tissue sections containing at least 70% tumoral cells using the QIAamp DNA Mini Kit (#51304, Qiagen K.K., Tokyo, Japan). DNA concentration was tested using Qubit Fluorometric Quantification (Thermo Fisher Scientific K.K., Tokyo, Japan). All cases were assessed for DNA quality by polymerase chain reaction (PCR) amplification following the BIOMED-2 guidelines [21].

The targeted NGS study was conducted using a custom gene panel that included 168 genes related to aggressive B-cell lymphomas and follicular lymphoma, based on the literature (Appendix A, Table A1). The NGS tech-workflow used the protocol SureSelectXT, 4 pools of 8 libraries (equimolar), 4 runs, MiSeq 2 × 131, and kit 300 cycles v3 [17].

The quality filtering of raw fastq NGS reads was performed using fastp version 0.20.0 with the default parameter. The filtered reads were mapped to the RefSeq Human Genome GRCh37.p13 using Burrows-Wheeler Aligner (version 0.7.12) to generate aligned BAM. Before somatic variant calling, the mapped files were manipulated using Samtools 1.16.1, Picard (http://broadinstitute.github.io/picard/; accessed on 10 April 2023), and GATK version 4.1.9 according to GATK best practices. The somatic variant calling was used with Mutect2 pipeline, HaplotypeCaller, FreeBayes (version 1.3.6), LoFreq (version 2.1.3.1), and VarScan2 (version 2.3). The identified variants were left aligned using bcftools version 1.18. The ANNOVAR software tool was used to functionally annotate the called variants.

Samtools is a set of utilities that manipulate alignments in the SAM (Sequence Alignment/Map), BAM, and CRAM formats. It converts between the formats, sorts, merging, and indexing, and can retrieve reads in any region swiftly. Picard is a set of command-line tools for manipulating high-throughput sequencing (HTS) data and formats such as SAM/BAM/CRAM and VCF. The GATK is the industry standard for identifying SNPs and indels in germline DNA and RNAseq data.

High confident calls were selected according to strict criteria in the following order:

1st. Variants identified by at least three programs, mutation caller (num.caller 2 to 5).
2nd. Exonic, exonic;splicing, and splicing calls (Func.refGene).
3rd. Allele Frequency VAF >3.5% (AlleleFreq > 0.035).
4th. Nonsynonymous mutations and damaging in at least 2 of 4 softwares (SIFT, Polyphen2_HVAR, mutation assessor, and CADD_phred). In SIFT, the calls that were selected were the “D” (damaging); in Polyphen2, the “D”; in Mutation Assessor, the “H” (high functional impact) and “M” (medium), the low and neutral (nonfunctional) were discarded; and in CADD phred ≥ 20.
5th. Avsp150 all sites ≤1% (i.e., avsp150 >1% were excluded).
6th. Not synonymous (ExonicFunc.refGene) (i.e., synonymous were excluded).

## 3. Case Report

This is a case report of a 74-year-old man who initially presented the development of a tumor in the neck. Relevant antecedents included long-term heavy smoking and stomach adenocarcinoma that was successfully treated by surgery 15 years ago. One year ago, he had a pathological fracture of the vertebra Th8–10 that was diagnosed with a probable metastasis from a previous stomach adenocarcinoma, treated with radiotherapy, and stabilized by an internal fixator.

Laboratory analysis showed anemia (RBC 3.96 × 10^6^/μL (reference values 4.35~5.55), Hb 11.4 g/dL (13.7~16.8), Ht 36.0% (40.7~50.1)), neutrophilia (81.2% (40~70)), lymphopenia (14.2% (20~40), eosinopenia (0.8% (1~4), hypoalbuminemia (4.0 g/dL (4.1~5.1), high alkaline phosphatase (143 U/L (38~113)), high BUN (urea nitrogen 22 mg/dL (8~20) hyperglycemia (124 mg/dL (73~109)), and hyperkalemia (high potassium) (5.1 mmol/L (3.6~4.8). The levels of C-reactive protein were normal (<0.09 mg/dL (0.00~0.14)). The patient was negative for HIV, hepatitis c, and hepatitis b viruses. Other parameters were normal. The patient was afebrile, no night sweats, and vitally stable. The physical examination of the neck demonstrated a right supraclavicular (lymph) node enlargement, confirmed with magnetic resonance imaging (MRI). A fine needle aspiration exploration was performed, but the cytological analysis was non-assessable. The patient underwent a tumor excision of the soft tissue (lymph node) of the neck.

**Figure 1:**
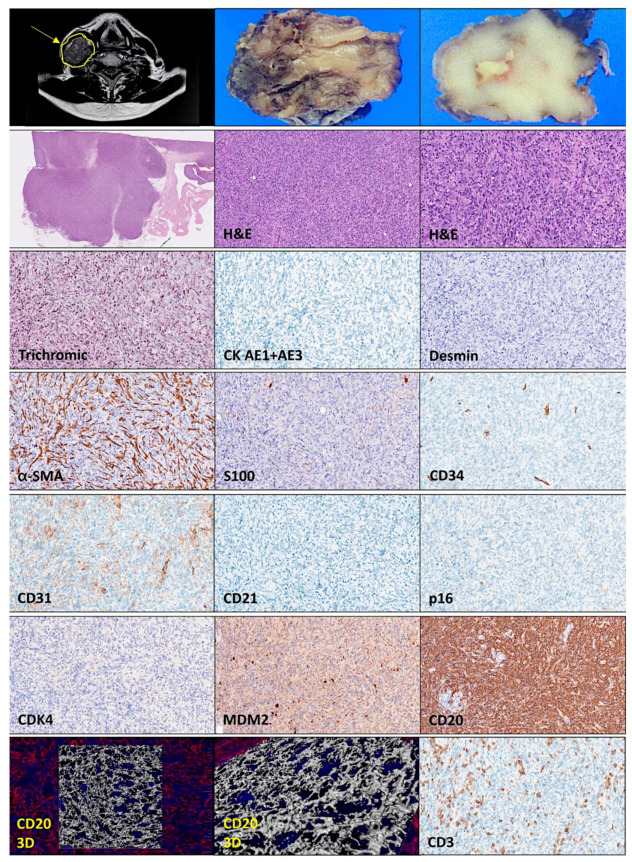
Histological features of the right supraclavicular (lymph) node enlargement. The histological features showed a diffuse proliferation of spindle cell cells that were MDM2- and CD20-positive. The spindle cell morphology was confirmed using confocal microscopy and 3D rendering.

**Figure 2:**
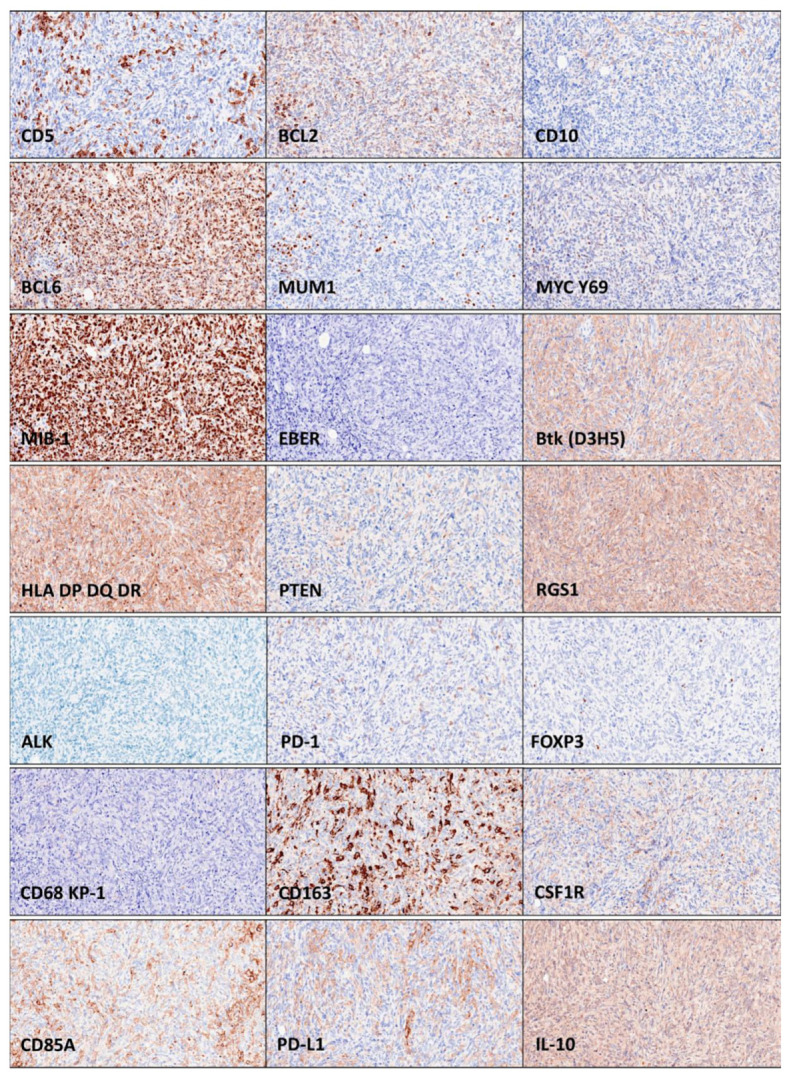
Histological features of the right supraclavicular (lymph) node enlargement (continuation). The neoplasia was BCL2-, BCL6-, MYC-, MIB-1-, BTK-, HLA-DP-DR-, RGS1-, and IL-10-positive. Being CD10-negative, BCL6-positive, and MUM1-negative, the spindle cell variant diffuse large B-cell lymphoma had a cell-of-origin of the germinal center B-cell (GCB) type. The immune microenvironment was characterized by moderate PD-1-positive cells, low FOXP3 Tregs, and moderate/high infiltration of tumor-associated macrophages (TAMs) with M2-like polarization as CD163, CSF1R, CD85A (LILRB3), and PD-L1 positivity.

**Figure 3:**
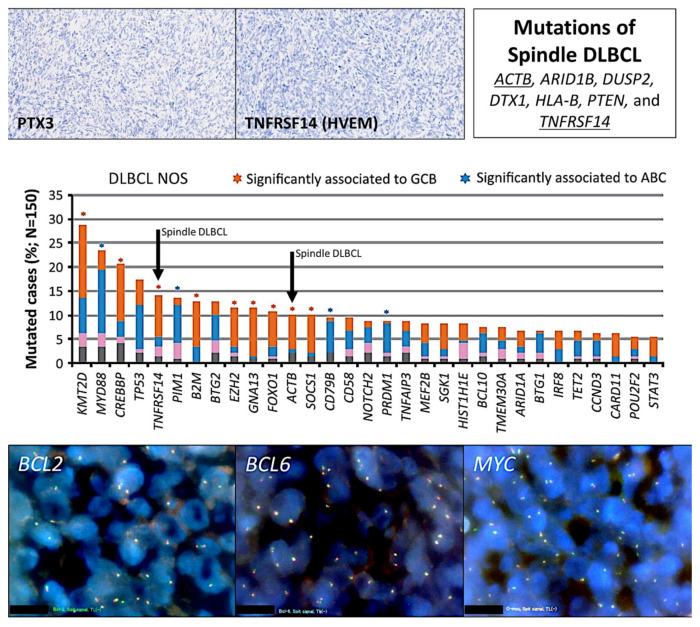
Histological features and mutational profile of the right supraclavicular (lymph) node enlargement. The protein expression of PTX3 and TNFRSF14 was negative. Mutational profiling using an in-house panel of 156 genes of aggressive B-cell lymphomas showed mutations of *ACTB*, *ARID1B*, *DUSP2*, *DTX1*, *HLA–B*, *PTEN*, and *TNFRSF14*. Notably, mutations of *TNFRSF14* and *ACTB* are usually found in GCB–type diffuse large B-cell lymphoma as described by Karube et al. [22]. Rearrangement of *BCL2*, *BCL6*, and *MYC* using DNA split probes were negative by FISH.

**Figure 4:**
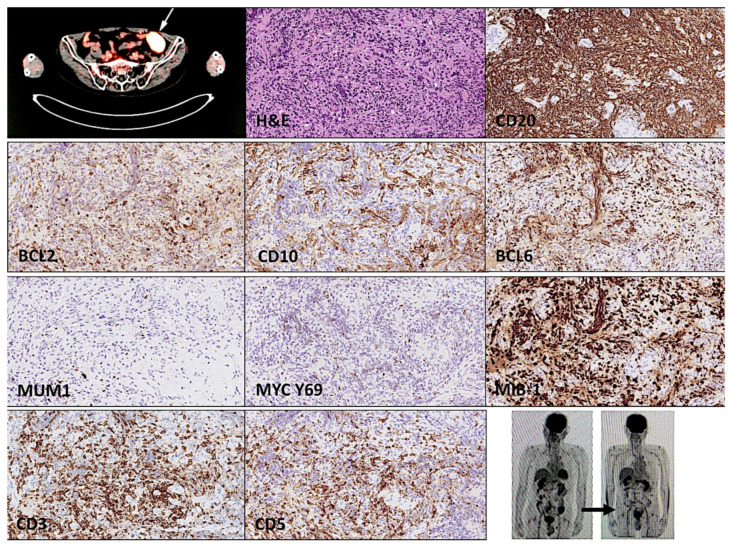
Histological features and mutational profile of the sigmoid mass. The sigmoid mass had the same immunophenotype as the right supraclavicular (lymph) node enlargement. It was CD20-positive, BCL2-positive, CD10-negative, BCL6-positive, and MUM1-negative. The expression of MYC was moderately positive, and had a high proliferation MIB-1 index. Infiltration of the immune microenvironment with CD3- and CD5-positive T lymphocytes was abundant. After treatment, the sigmoid mass shrunk.

**Figure A1:**
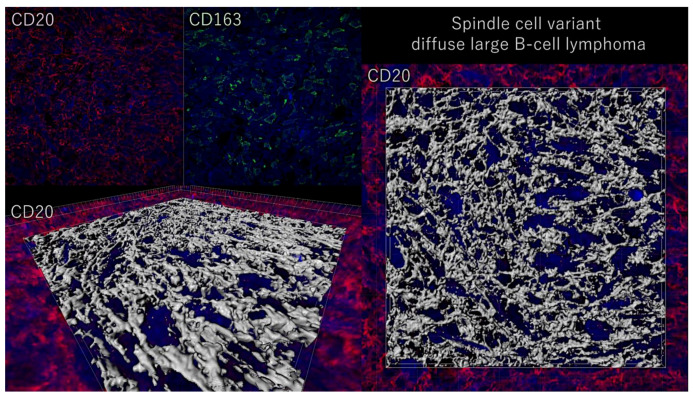
Three-dimensional rendering of spindle cell variant diffuse large B-cell lymphoma.

Figure 1, Figure 2, Figure 3 and Figure 4 show all the clinicopathological characteristics, and in the Appendix A (Figure A1), additional 3D images are shown. Gross examination of the specimen revealed a partially circumscribed, loosely capsulated node of 50 × 33 × 26 mm that, at sectioning, had a pale white color and was soft to the touch. Microscopic evaluation using hematoxylin and eosin (H&E) staining led to the identification of residual peripheral lymph node tissue and showed that the lymph node was effaced by a diffuse proliferation of large cells with scan cytoplasms, multiple small nucleoli, and infrequent mitotic activity. Interestingly, most of the cells had a spindle cell morphology (long and slender). Trichromic staining visualized a moderate presence of connective tissue. Immunohistochemical analysis using anti-pan cytokeratin (AE1 + AE3) antibody to search for epithelial differentiation was negative. Staining for desmin, an intermediate filament protein of both smooth and striated muscles, was also negative. Alpha smooth muscle actin antibody (α-SMA) stained the smooth muscle cells in the vessel walls, but also highlighted a dense network of cancer-associated fibroblasts (CAFs). The S100 staining also weakly highlighted the fibroblasts and macrophages, but also showed the occasional presence of S100+ cells of the neuro-immune cross-talk in lymph organs. Endothelial cells were highlighted with CD34 and 31 staining, but the tumoral cells were negative. CD31+ tumor-associated macrophages were also identified. In the peripheries, CD31+ monocytoid cells were found, as well as CD31+, CD3+, and PD-1+ T cells. The CD21 staining only highlighted the follicular dendritic cells of the germinal centers of lymphoid follicles in the residual peripheral lymph node tissue. The spindle cell tumoral cells were p16- and CDK4-negative, but weakly positive for MDM2. Notably, CD20, which is a common B-cell marker also called L26, a membrane spanning four domains (MS4A1), was strongly and diffusely positive.

Because of the histological features and the positivity for CD20, the case was diagnosed as DLBCL, spindle cell variant. The histological features of the B-lymphocytes were further analyzed using immunofluorescence for CD20 and confocal microscopy with 3D rendering. The 3D reconstruction confirmed the spindle cell morphology. The diagnosis was confirmed by further immunophenotype characterization (protein expression/levels). The tumoral B-lymphocytes had an immunophenotype that was CD3−, CD5−, CD20+, and BCL2+. The cell-of-origin classification using Hans’ algorithm was germinal center B-cell-like (GCB-like), being CD10−, BCL6+, and MUM1−. The expression of the MYC oncogene was low (20–40%). The tumor had a high proliferation index (MIB-1 > 90%). The Epstein–Barr virus-encoded RNA in situ hybridization (EBER-ISH) was negative. The protein expression of Bruton’s tyrosine kinase (Btk) was strong/moderate and diffuse. The expression of the class II molecules HLA-DP, DQ, and DR was strong/moderate. The expression of MDM2, an oncogene inhibitor of p53 and positive regulator of the cell cycle, was diffusely moderate/weak. The expression of the tumor suppressor PTEN was negative (only positive in stromal cells). The protein expression of RGS1, a regulator of chemotaxis and associated with poor prognosis of DLBCL [8], was strong. ALK expression was negative.

Immuno-oncology markers of the immune checkpoint and microenvironment were also analyzed. The infiltration of CD3+ and CD5+ T lymphocytes was low (<10%), PD-1-positive cells were scarce (<5%), and FOXP3+regulatory T lymphocytes (Tregs) were almost absent (<1%). The pan-macrophage marker CD68 (KP-1) highlighted high infiltration of tumor-associated macrophages (TAMs) (>10%−20%). These TAMs had a pro-tumoral M2-like macrophage polarization [9], being CD163+, CSF1R+, CD85A+, and PD-L1+. The analysis of the M2c-like immune regulatory phenotype showed positivity by TAMs and tumoral B-lymphocytes for IL-10, but negativity for PTX3. The TNFRSF14 (HVEM) expression was negative.

Molecular characterization using DNA split probes did not show a rearrangement of the *MYC*, *BCL2*, and *BCL6* genes (i.e., translocation negative) (Figure 3). The mutational landscape was performed using next-generation sequencing (NGS) and a custom-made panel of 156 genes related to aggressive B-cell lymphomas (DLBCL) and follicular lymphoma (Appendix A). After filtering for the high confidence calls, the mutated genes were *ACTB* (damaging non-synonymous SNV), *ARID1B* (non-frameshift deletion), *DUSP2* (damaging non-synonymous SNV), *DTX1* (stop-gain), *HLA-B* (frameshift insertion and deletion), *PTEN* (stop-gain), and *TNFRSF14* (damaging non-synonymous SNV).

Based on the NIH National Cancer Institute LymphGen 1.0 classification tool (internet link: https://llmpp.nih.gov/lymphgen/lymphgendataportal.php; accessed on 28 February 2023), this case had a ST2 subtype prediction. Since *NOTCH1* mutations were not available, the prediction of the N1 subtype was excluded.

Just after the scission of the neck tumor, the patient underwent further clinical evaluation. The variables necessary for calculating the International Prognostic Index (IP) were age > 60 years (one), ECOG performance status zero–two (zero), LDH normal (zero), extranodal sites zero–one (one), and clinical stage IVAE (one). Therefore, the revised DLBCL IPI (R-IPI) had a score of two and the prognosis was good, with a predicted 4-year progression-free survival rate of 80%, and estimated overall survival of 79%. After surgery, positron emission tomography and computed tomography (PET/CT scan) highlighted an additional sigmoid tumoral mass. A biopsy of the sigmoid mass (Figure 4) confirmed a DLBCL diagnosis that had the same histological features as the nodal tumor of the neck. Although the biopsies were small (2 × 2 × 1 mm) and had a biopsy artefact, cells with spindle cell morphology could be identified. The immunophenotype was CD20+, BCL2+, CD10−, BCL6+, MUM1−, MYC low, and MIB-1 high. The EBER was negative, and the ISH for kappa and lambda did not show restriction. CD3 and CD5 were both negative.

The patient underwent five cycles of R-CHOP therapy with a metabolically complete response. A PET/CT scan confirmed the disappearance of the sigmoid mass (Figure 4). After a follow-up of 2 years and 3 months, he is still alive.

## 4. Discussion

This is a case report of a spindle cell diffuse large B-cell lymphoma, which is a very rare morphological variant. The diagnostic key was the positivity for CD20. Nevertheless, in the diagnosis, other spindle cell lesions must be considered. Spindle cell lesions of the head and neck include several types of benign tumors and malignant neoplasia, and can be a diagnostic challenge for pathologists. The use of immunohistochemical markers can help differentiate the origin of the different types of neoplasms, including neural, myofibroblastic, muscular, fibroblastic, vascular, epithelial, odontogenic, and miscellaneous [10]. The differential diagnosis is large. For instance, neural tumors include neurofibroma, neurilemmoma (schwannoma), palisade-encapsulated neuroma, and malignant peripheral nerve sheath tumor (MPNST); useful markers for these diagnoses include S100, EMA, CD57, and collagen IV. Myofibroblastic tumors such as the myofibroma and myofibrosarcoma are usually vimentin-, SMA- and desmin-positive, but also focally positive for epithelial markers such as CK and EMA. Muscle tumors such as leiomyosarcoma are desmin-, vimentin-, SMA-positive. Fibroblastic tumors such as fibrosarcoma are vimentin- and CD99-positive, but negative for muscular markers (desmin, SMA), osteoblasts (osteocalcin), macrophages (CD68), neural tissue (S100), hematopoietic cells (CD34), and epithelial tissue (CK, EMA). Vascular tumors include Kaposi sarcoma and spindle cell hemangioma, and vascular markers such as CD31 and CD34 are useful. The detection of HHV-8 LNA-1 and D2-40 are useful for Kaposi sarcoma diagnosis. Epithelial tumors such as spindle cell carcinoma are keratins (CK AE1/AE3) and EMA. Malignant melanoma is typically positive for vimentin, S100, HMB-45, melan-A, and MITF. Markers for desmoplastic melanoma are p16, WT-1, SOX-10, nestin, and p75 [23]; the markers that allow the differentiation of spindle cell melanoma versus desmoplastic melanoma are laminin, p75, HMB-45, c-kit (CD117), melan-A, col IV, CD68, and MDM2 [24]. Gastrointestinal spindle cell tumors (GIST) are c-kit- (CD177), DOG-1-, PKC-theta-, and CD34-positive [25,26,27,28]. Finally, in the miscellaneous tumors, benign and malignant fibrous histiocytoma must be considered, with the positivity of vimentin and CD68; and synovial sarcoma with TLE1 positivity [29]. Table 1 summarizes the differential diagnoses.

There are several possible diagnoses. The head and neck region, poses a critical diagnostic challenge because of the overlapping spectrum of clinico-radiologic and microscopic features of the tumors. The use of immunohistochemical markers can help differentiate the origin of the different compatible neoplasms, including neural, myofibroblastic, muscular, fibroblastic, vascular, epithelial, odontogenic, and miscellaneous [10]. In the sigmoid mass, the differential diagnosis also includes gastrointestinal stromal tumors (GIST). In Table 1, DLBCL stands for diffuse large B-cell lymphoma; + stand for positive in the immunohistochemistry; − stands for negative; and +/− stands for positive or negative.

**Figure A2:**
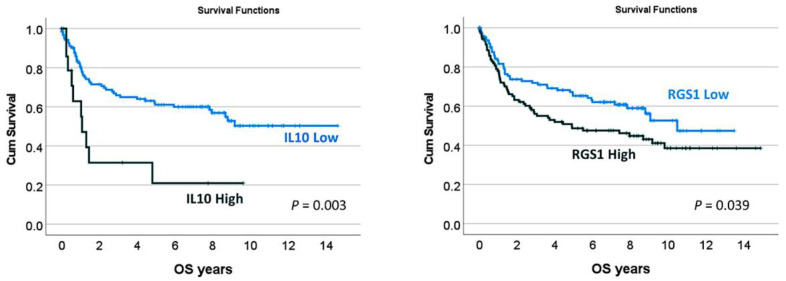
Prognostic values of IL-10 and RGS1 in DLBCL.

A few cases of extranodal B-cell lymphoma have been reported. Yiting Li et al. reported two cases of diffuse large B-cell lymphoma [30]. Both cases were treated with R-CHOP therapy with a favorable clinical response. The second case was a GCB subtype based on the Hans’ classifier of cell-of-origin, and NGS analysis revealed gene alterations in *EZH2*, *IRF8*, and *TNFRSF14*. Notably, our case was also GCB and had a *TNFRSF14* mutation. J Wang et al. reported five cases of diffuse large B-cell lymphoma with spindle cell components involving the skin, nasal-ocular, and soft tissue [31]; however, the immunophenotype specific to lymphoma was limited. Another case has recently been reported by Toprak S et al. that was CD45-, CD30-, and PAX5-positive [13]. Finally, a larger series of cases were reported by Kimura Y [12] and Ohshima K et al. with 10 cases; these cases were characterized by GCB origin, were EBER-negative, and did not have any other specific phenotype or any karyotypic abnormalities, including the absence of *BCL2*, *BCL6*, and *MYC* translocations [12]. Interestingly, T-cell/myofibrohistio-rich stromal alterations were noted, similar to our case, that had moderate infiltration of tumor-associated macrophages (TAMs). In our case, the TAMs had an M2-like polarization, with positivity of CSF1R, CD163, and IL-10 that is compatible with an M2c-like subtype of immune regulatory properties. Additionally, the tumor was IL-10 and RGS1, markers that we have described to be associated with poor prognosis in diffuse large B-cell lymphoma (Figure A2) [18,19,32,33,34].

Based on the LymphGen 1.0 classification tool, this case had an ST2 subtype prediction [35]. Based on the work of Wright et al., diffuse large B-cell lymphoma consisted of seven genetic subtypes. The LymphGen algorithm can classify the cases into one or more genetic subtypes that have distinct clinical outcomes and pathway dependencies. It is expected that these genetic subtypes will help the development of targeted therapy [35]. Our case had an ST2 subtype that corresponds to the GCB cell-of-origin subtype and is associated with favorable prognosis [35].

The strengths of this case report are the thorough immunophenotype characterization and the custom-made NGS panel. The fact that only one case is reported is a limitation. In the future, a larger series of cases will be necessary to characterize this morphological variant.

In conclusion, we described a case of spindle cells diffuse large B-cell lymphoma of the GCB phenotype, which was EBER-negative, had positivity for IL-10 and RGS1, with a microenvironment rich in TAMs, and a *TNFRSF14* mutation.

## Figures and Tables

**Table 1:** Differential diagnosis.

Marker	Leiomyosarcoma	Angiosarcoma	Fibrous Histiocytoma	Spindle Cell Squamous Cell Carcinoma	Desmoplastic Melanoma	Spindle Cell Variant DLBCL
SMA	+			+/−		−
Desmin	+				+/−	−
CD34		+				−
S100						−
Endothelial (CD31)		+				−
Melanocytic(HMB-45, Melan–A)					+/−	−
Epithelial(CK AE1/AE3, EMA)	+/−	+/−		+	+/−	−
Macrophage markers (CD68)			+	−	−	−
B-cell marker (CD20)	−	−	−	−	−	+

## Data Availability

All data are available upon request to Joaquim Carreras (joaquim.carreras@tokai-u.jp). The raw images are uploaded into the CERN OpenAIRE repository (https://zenodo.org; accessed on 10 March 2023): Carreras, Joaquim. (2023). spindle cell variant diffuse large B-cell lymphoma (hematolrep-15-00020) (Version 1). Zenodo. https://doi.org/10.5281/zenodo.7682045; accessed on 10 March 2023). Additionally, the excel file with the high confidence calls has also been uploaded in the same Zenodo platform: Carreras, Joaquim. (2023). spindle cell variant diffuse large B-cell lymphoma (NGS annotation file; high confidence calls) hematolrep-15-00020 (Version 1) [Data set]. Zenodo. https://doi.org/10.5281/zenodo.7682122; accessed on 10 March 2023).

## Appendix A

**Table A1:** List of genes in the custom NGS panel.

*ACTB*	*ARID1A*	*ARID1B*	*ARID5B*	*ATM*	*ATP10A*	*B2M*	*BCL10*	*BCL2*	*BCL6*
*BCL7A*	*BCOR*	*BLNK*	*BRAF*	*BTG1*	*BTG2*	*BTK*	*CARD11*	*CCL22*	*CCND3*
*CD22*	*CD274*	*CD276*	*CD28*	*CD36*	*CD40*	*CD40LG*	*CD58*	*CD70*	*CD79A*
*CD79B*	*CD80*	*CD83*	*CD86*	*CDKN2A*	*CDKN2B*	*CIITA*	*CREBBP*	*CSF1R*	*CTLA4*
*CXCR4*	*CXCR5*	*DDX3X*	*DIS3*	*DTX1*	*DUSP2*	*EBF1*	*EP300*	*ETS1*	*ETV6*
*EZH2*	*FAS*	*FBXW7*	*FOXO1*	*GNA13*	*GNAI2*	*HAVCR2*	*HIST1H1B*	*HIST1H1C*	*HIST1H1D*
*HIST1H1E*	*HIST1H3G*	*HLA-A*	*HLA-B*	*HLA-C*	*HVCN1*	*ICOS*	*ICOSLG*	*ID3*	*IDO1*
*IKZF1*	*IL10*	*IRF2BP2*	*IRF4*	*IRF8*	*ITPKB*	*JUNB*	*KLHL14*	*KLHL6*	*KMT2D*
*KRAS*	*LAG3*	*LGALS9*	*LPHN2*	*LYN*	*MALT1*	*MAML1*	*MAP2K1*	*MAPK1*	*MCL1*
*MEF2B*	*MFHAS1*	*MYC*	*MYD88*	*NFKB1*	*NFKB2*	*NFKBIA*	*NFKBIE*	*NFKBIZ*	*NOTCH1*
*NOTCH2*	*NRAS*	*OSBPL10*	*P2RY8*	*PARP2*	*PAX5*	*PCBP1*	*PDCD1*	*PDCD1LG2*	*PIK3CA*
*PIK3CD*	*PIK3R1*	*PIM1*	*PLCG2*	*PLOD2*	*POU2AF1*	*POU2F2*	*PRDM1*	*PRKCB*	*PTEN*
*PTPN6*	*PTPRD*	*REL*	*RELA*	*RGS1*	*RHOA*	*RRAGC*	*S1PR1*	*S1PR2*	*SETD1B*
*SETD2*	*SF3B1*	*SGK1*	*SMARCA4*	*SOCS1*	*SPEN*	*SPIB*	*STAT3*	*STAT6*	*SYK*
*TBL1XR1*	*TCF3*	*TET2*	*TGFB1*	*TMEM30A*	*TMSB4X*	*TNFAIP3*	*TNFRSF14*	*TNFRSF18*	*TNFRSF4*
*TNFRSF9*	*TNFSF18*	*TNFSF4*	*TNFSF9*	*TNIP1*	*TOX*	*TP53*	*TP53BP1*	*TP63*	*TREML2*
*TYRO3*	*UBE2A*	*VSIR*	*VTCN1*	*XBP1*	*XPO1*	*ZEB2*	*ZFP36L1*		

The neoplastic CD20-positive B-lymphocytes of diffuse large B-cell lymphoma have spindle cell morphology, which is a very infrequent morphological variant.

We recently described the prognostic value of the immunohistochemical expression of IL-10 and RGS1 in diffuse large B-cell lymphoma [18,19,32]. These two markers are associated with high infiltration of immune regulatory tumor-associated macrophages (IL-10-positive TAMs), and to 1q copy-number gain, respectively. This spindle cell variant case was also positive for these two markers. The clinical relevance of this finding should be explored in a larger series of spindle cell cases.
